# Supplementary material for: Are carbon emissions trading and green financial instruments synergistic? -Comprehensive quantitative research based on content analysis
Source: PLoS One. 2024 Mar 7;19(3):e0298601. doi: 10.1371/journal.pone.0298601 (PMC10919601; doi:10.1371/journal.pone.0298601)
Supplement: S1 Data — (ZIP) [file pone.0298601.s001.zip › Data/地级市Z_F工作报告.zip!/╔╜╢1⁄2/═■║ú╩╨/└┤╫╘╣1⁄2╓┌║┼í╛┬φ┐╦╩2╛▌═°í┐_2007.docx]

各位代表：

现在，我代表市人民政府，向大会作政府工作报告，请予审议，并请各位政协委员和其他列席会议的同志提出意见。

一、2006年政府工作的回顾

过去的一年，在中共威海市委的正确领导下，市政府团结和带领全市人民，坚持以邓小平理论和“三个代表”重要思想为指导，深入贯彻党的十六大和十六大以来的各次中央全会精神，认真落实市委“一一六一”发展目标和思路，按照“五个三”的工作要求，全面强化了落实科学发展观、构建社会主义和谐社会的各项工作及其考核办法，完成了市十四届人大四次会议确定的主要目标任务，实现了“十一五”的良好开局，经济和社会都发生了新的变化。

――全市生产总值达到1368.5亿元，比上年增长15.9%；财政总收入完成131.9亿元，比上年增长19.9%，其中地方财政收入70.1亿元，比上年增长21.8%，经济发展的质量和效益都有新的提高。

――全市万元生产总值能耗、水耗预计均降低3%，化学需氧量和二氧化硫排放量预计分别削减8%和1.2%，经济增长方式更加集约。

――全市社会消费品零售总额增长16.3%，固定资产投资增长17.9%，外贸出口增长27%，经济运行更加协调。

――全市城镇居民人均可支配收入达到13975元，农民人均纯收入达到6842元，分别增长12.2%和12.5%，人民生活更加殷实。

――全市用于统筹城乡发展、公益事业和社会保障的财政投入达到51.1亿元，增长22.3%，城镇登记失业率控制在1.6%以内，社会发展更加和谐。

一年来，我们主要做了以下十个方面的工作：

（一）坚决执行中央宏观调控政策，推动了经济平稳协调发展。按照中央和省里要求，我们强化了建设用地的审批管理和集约利用考核，优化了金融生态环境，加强了投资管理和新上项目清理。全年处置盘活闲置土地1200多亩，查处新开工违规项目10个，因环境因素拒批建设项目33个。固定资产投资完成697亿元，增幅比上年回落7个百分点。投资结构进一步优化，农业、高新技术产业和服务业的投资分别增长20.6%、51%和19.2%，产能过剩行业投资得到有效遏制，经济发展的稳定性和协调性明显增强。

（二）狠抓关键环节，加快了经济增长方式的转变。加强了威海国际生物技术研发中心、威海国际微电子技术中心、宋健科学技术研究院、威海火炬创新创业基地等创新载体建设，新建了5个科技创新公共研发平台，加大了对161个中小企业科技创新项目和273家在孵企业的扶持力度。新建市级以上工程研究中心9家、企业技术中心17家，威高集团技术中心被认定为国家级企业技术中心。高新技术产业产值占工业总产值的比重由24.9%提高到27%。全年引进各类人才6616名，取得重要科技成果115项，专利授权量增长43.4%。循环经济快速发展，有24家企业通过了清洁生产审核。生态市建设扎实推进，生态示范区建设试点顺利通过国家验收；荣成、文登、乳山在全省率先通过了创建国家生态县省级考核；中心城市环境综合整治定量考核连续8年位居全省第一，在生态省建设目标责任中期考核中被评为先进市。

（三）深化结构调整，促进了产业结构的优化升级。深入推进了“三大基地”建设，三次产业结构由上年的9.2：62：28.8调整为8.5：62.1：29.4。产业集群加速膨胀，三星、伽耶、大宇等造船项目相继落户，华泰、哈飞、广泰等汽车项目获得批准，五大产业群对工业增长的贡献率达到88.8%。新增中国名牌产品8个、山东名牌产品25个、山东著名商标12个。刘公岛空中快车、温泉开发等重点旅游项目正式启动，旅游大篷车、诚信旅游等活动取得新成效，旅游总收入增长19%。成功举办了第三届国际人居节、东亚投资论坛、东北亚机械产业城市联合第二届市长会议及14个专业展会。加快推进了中心商业区改造。新建“农家店”630多处，我市被商务部确定为全国“万村千乡市场工程”示范点。

（四）统筹城乡发展，推进了社会主义新农村建设。积极落实各项惠农政策，全市财政支农资金达到13.8亿元，增长25.1%。粮食总产达到96.3万吨，增长1.4%；主要经济作物和畜产品稳定增产，现代海洋产业稳步发展，农林牧渔业增加值达到116.6亿元，增长4.6%。在全省提前对农村义务教育阶段学生免收了学杂费。新型农村合作医疗覆盖率达到95.3%，44.7万农民享受到了医疗补助。在全省率先基本实现村村通自来水，普及率达到95.3%。实施了“百村示范、千村整治”工程，建成152个文明生态村，43个镇被评为全国环境优美乡镇，占到全省总数的34.7%。

（五）抢抓战略机遇，提高了全方位开放的层次和水平。成功组织了赴美国、韩国、西安、北京、哈尔滨等30多次国内外经贸推介活动，引进了56个投资过千万美元的外资项目、82个过亿元的内资项目，实际利用外资和内资分别增长7.9%和20%。“走出去”战略取得历史性突破，新办境外企业6家，外派劳务6500人。进出口总值达到95.1亿美元，增长26.1%。9个开发区通过了国家审核，威海工业园、荣成工业园、文登工业园被批准为省级开发区。外事、侨务、对台和国际友城工作也取得了新成绩。

（六）深化各项改革，进一步激发了体制机制活力。健全了国有资产监管机制，加强了机关事业单位资产收益管理。深化了国库集中支付和部门预算制度改革，完善了社保基金、住房公积金监管体制。开展了财税执法大检查，加强了税源动态管理。推进了蓝星玻璃资产重组、国铁控股我市地方铁路的相关工作，完成了园林处等市政单位的转企工作。广泰空港成功在深交所上市，万丰奥威实现间接上市。出台了加快金融业发展和创新的政策措施，深化了农村信用社改革，在中央银行票据兑付工作上取得突破。

（七）强化精品意识，优化了人居环境。编制完成了城乡统筹规划、临港工业园发展等专业规划，推进了城市管理相对集中行政处罚权工作。加快了威乳高速及疏港路、机场改造等基础设施工程建设，开通了威海至徐州的旅客列车，机场旅客吞吐量突破40万人次。中心市区开工建设了威海新外滩、悦海公园等重点工程；加快推进了城中村改造，对7个居民小区、6条主要河流和威海湾进行了环境综合整治；新建改造供排水管道360公里，第三污水处理厂正式投入运营。荣成市市民广场改造、绿岛湖公园建设等工程，文登市新城中心区建设、河道整治等工程，乳山市文化博览中心、中心绿廊等建设工程扎实推进，打造了一批精品工程，城市承载功能都有新的提高。全市完成植树2800万棵，森林覆盖率提高了2个百分点。被评为“全国绿化模范城市”、“中国水环境治理范例城市”，被命名为“中国温泉之乡”。

（八）关心群众生活，解决了一批事关群众利益的实际问题。在全省率先推行了城乡统筹就业和城镇医疗保险全覆盖试点，提高了最低工资标准，企业养老、医疗保险参保人数分别增加3.3万人和6.3万人。修订了市区中小学建设的长远规划，完成了城里中学搬迁和威海四中扩建等工程，新增校舍6.9万平方米，缓解了班额过大的问题。推进了学生营养餐工程，在鲸园小学等3处地段建设了过街天桥或地下通道。加强了社会救助工作，城乡最低生活保障标准分别提高到每人每月300元和每人每年1000元，农村五保集中和分散供养标准分别提高到2400元和1400元，对12325名贫困家庭学生进行了专项资助，对2300多名残疾人实施了光明、助听、助行及扶持就业等行动，对4000多名困难群众进行了及时救助。

（九）注重统筹兼顾，强化了和谐威海建设。基本完成了公共卫生“两个体系”建设，加强了75个社区卫生服务中心和256个农村标准化中心卫生室建设，顺利通过了国家卫生城市复检。完成了人口和计划生育工作的各项目标，对1.5万人发放了农村计生奖励扶助费。文化事业获得长足发展，《梦海》剧目在省文博会汇演中获得特别贡献奖，并应邀赴韩国、法国、新加坡等国家演出，扩大了威海在海外的影响。全民健身活动更加普及，取得21届省运会金牌总数第六名的好成绩。对口支援和重点帮扶工作取得了积极成效。广播电视、新闻出版等事业繁荣活跃，在全省率先实现了有线电视村村通。市志编修和档案资源挖掘开发工作扎实推进。军政军民关系更加巩固。民族、宗教、妇女儿童、红十字、气象、人防、地震等事业取得新的成绩。文明威海、诚信威海建设扎实推进，精神文明建设取得新进展。民主法制建设得到加强，“五五”普法顺利启动，平安威海建设深入推进，刑事案件、安全事故数量分别下降3.9%和19.9%，“三市一区”全部进入全省平安建设先进县行列。

（十）坚持依法行政，加快了政府职能的转变。认真实施《公务员法》，加快推进了政府管理创新。建立和推行了行政执法责任制，规范了部门行政审批服务窗口，镇和街道办事处全部建立了便民服务中心，在全省率先完成了三级行政审批服务体系建设。健全了突发公共事件应急管理体系。认真办理了人大代表建议和政协委员提案，及时向人大汇报工作、向政协通报情况。在全省率先设立了社情民意调查中心，加强了民主评议行风工作，完善了“早上听广播、白天打热线、晚上看直播、全天发邮件”的多元沟通渠道。严肃查处了违法违纪案件，廉政建设和反腐败工作取得新的成效。

各位代表！建设人民满意的政府，是我们不懈的追求。社情民意调查中心对全市400户家庭的一项随机调查显示，88%的市民对市政府过去一年的工作感到满意。这一评价，对我们来说，既是鼓舞，更是鞭策。我们深知，亲民、为民、富民，是政府的职责所在。要建设一个人民满意的政府，必须树立科学的发展观，聚精会神搞建设、一心一意谋发展；必须树立正确的政绩观，踏踏实实地为群众办实事、解难事、做好事；必须树立辩证的和谐观，更加关注民生问题，让人民群众在改革和发展中得到更多实惠；必须树立正确的权力观，做到权为民所用、情为民所系、利为民所谋，自觉接受人民群众的监督。我们一定遵循这些要求，认真履行职责，不辜负人民群众的信任和期望。同时，我们也深知，政府工作所取得的每一份成绩，无不凝聚着同志们、朋友们的心血和汗水。在此，我代表市人民政府，向在各个领域和岗位上辛勤劳动、无私奉献的全体市民和所有纳税人，向给予政府工作热情支持的人大代表和政协委员，向各民主党派、工商联、各人民团体和社会各界人士，向驻威部队指战员、武警官兵和政法干警，向各位老领导、老同志，向所有关心支持威海建设的海内外朋友，致以崇高的敬意和衷心的感谢！

各位代表！在总结成绩的同时，我们也清醒地看到，我市经济社会发展中还存在一些矛盾和问题，政府工作中也还有许多缺点和不足。主要是：经济结构性矛盾依然突出，投资和消费关系不够协调，服务业发展还相对滞后；粗放型经济增长方式仍未得到根本转变，自主创新能力还不够强，资源利用效率还有待进一步提高，生态环境保护的压力依然很大；制约发展的体制机制障碍还比较多；收入分配中的矛盾仍比较突出，农民增收难度较大，社会保障制度不够完善；依法行政的能力和水平有待进一步提高，工作作风、行政效能与发展的要求还有一定差距，腐败现象和不正之风还时有发生，等等。对此，我们一定会高度重视，采取有效措施，逐步加以解决。

二、2007年政府工作的总体要求和目标

各位代表，2007年，党的十七大和市十三次党代会将要召开，同时又是地级威海市成立20周年。做好今年的工作，意义重大。政府工作的总体要求是：以邓小平理论和“三个代表”重要思想为指导，以科学发展观统领全局，认真贯彻党的十六大和十六大以来的各次中央全会精神，全面落实市委“一一六一”发展目标和思路，着力调整经济结构和转变经济增长方式，着力加强资源节约和环境保护，着力推进改革开放和自主创新，着力促进社会发展和解决民生问题，努力实现速度、质量、效益相协调，消费、投资、出口相协调，人口、资源、环境相协调，推动经济社会又好又快发展。

全市经济社会发展的主要预期目标为：生产总值增长13%，万元生产总值能耗、水耗分别降低4.5%和4%，化学需氧量、二氧化硫排放量分别削减7.5%和1.5%，地方财政收入增长16%，固定资产投资增长15%，社会消费品零售总额增长15%，外贸出口总值增长16%，实际利用外商直接投资增长8%，实际利用内资增长10%，城镇居民人均可支配收入增长10%，农民人均纯收入增长8%，城镇登记失业率控制在2%以内，人口自然增长率控制在 1‰以内。完成上述目标，要突出把握好以下四个方面：

一是更加自觉地贯彻中央宏观调控政策。把宏观调控作为调整优化结构、转变增长方式的重大机遇，用心把握政策取向，严把土地、信贷闸门和市场准入门槛，突出重点、区别对待、有保有压，努力消除发展中的不健康不稳定因素，防止大起大落，保持良好发展态势。

二是更加自觉地坚持又好又快发展。深刻领会“好”与“快”的科学内涵，不断转变政府的施政理念，突出发展的质量、效益和后劲，突出节能降耗、环境保护和自主创新，自觉地把“好”放在首要位置，坚持以“好”来统领“快”，在“好”中求“快”，不断提高经济增长的稳定性、协调性和可持续性。

三是更加自觉地维护社会和谐稳定。坚持以人为本，关注社会和民生，在社会管理和服务中体现人文关怀，维护公平正义，增进团结和睦，切实做到以改革促进和谐，以发展巩固和谐，以稳定保障和谐。

四是更加自觉地推进工作创新。坚持把创新贯穿于政府工作的各个方面、各个环节，自觉地以观念更新促创新，以改革开放促创新，以转变职能促创新，善于做好结合的文章，善于用创新的机制体制规范行为、推动工作，用发展的眼光、创新的思维、和谐的理念，不断研究新情况，解决新问题，在创新中创业创优，不断开创工作新局面。

三、推进经济又好又快发展

（一）加快推进经济增长方式的转变。要突出抓好两项重点工作：一是以节能降耗为重点，加快推进节约型社会建设。大力发展循环经济和清洁生产，重点抓好三角集团轮胎翻新、大业金矿尾矿开发等资源节约综合利用项目，培育10个循环经济、10个清洁生产示范企业。积极推广先进技术和节能设备，鼓励使用沼气、太阳能等可再生能源，鼓励发展节能节地型建筑，鼓励支持再生资源回收利用网络体系建设。深化资源性产品价格改革，落实好阶梯式水价，鼓励节约用水，中心市区搞好二热电、西郊热电厂海水综合利用和三污中水回用等工程，新建4座中水加水站点，铺设中水管网13.7公里。加强土地审批管理，严格落实投资强度、建筑容积率和建筑密度标准，对达不到标准的不予供地或核减用地面积。进一步整顿和规范矿产资源开发秩序，推进温泉资源的集约化综合开发。大力倡导绿色消费和节约理念，加大节能执法、节能审计、节能考核力度，确保完成各项约束性指标。

二是以自主创新为中心环节，大力推进创新型城市建设。强化科技创新体系建设，继续加大对宋健科学技术研究院等现有创新平台的扶持力度。加强大型科学仪器共享平台建设，优化科技服务环境。年内新增市级以上企业技术中心10家、工程研究中心7家。安排中小企业科技创新资金和科技三项经费4100万元，组织实施市级以上科技计划180项。围绕电子信息、新材料、新能源、光机电一体化、生物医药等五大领域，抓好碳纤维材料及制品、蓝星光伏发电等110个重点产业化项目，高新技术产业产值比重提高2个百分点。统筹规划全市的信息化建设，最大限度实现网络互通、资源共享。加快推进数字威海地理空间框架与应用示范项目建设，保障经济社会发展对基础地理信息的需求。积极做好国家知识产权试点城市、试点园区的各项工作，鼓励发明创造，争取专利申请量增长10%以上。加快高层次人才和各类急需专门人才的培养和引进，提高自主创新的能力和水平。

（二）大力调整优化产业结构。立足“三大基地”建设，加快构建更具竞争力的现代产业体系。

围绕现代制造业基地建设，加快推进新型工业化进程。一是加快发展产业集群。落实好近年来的招商成果，突出抓好大宇、伽耶等造船项目以及华泰、东安黑豹等汽车项目，加快形成集群效应。抓好北洋电子制签机、华力高效电机、威高医用新材料等115个重点工业项目建设，引导中小企业发展配套产业，进一步拉长产业链，壮大五大产业群。二是着力推动骨干膨胀。坚持抓大扶小，突出抓好60户市级重点企业和40户成长型、潜力型的中小企业，扶持优势企业加快发展。三是积极实施品牌战略。启动新一轮名牌争创工作，选择一批带动力强、关联度大、技术含量高的产品进行重点培植，力争在争创国家级和省级名牌上再有新突破。

围绕旅游度假业基地建设，大力发展现代服务业。一是加快旅游业发展。把旅游业作为服务业的龙头产业，全面实施“幸福海岸专项规划”，深度开发和整合资源，切实抓好刘公岛空中快车、温泉度假村、大乳山休闲度假区等重点项目建设。积极开拓海内外市场，搞好游艇、垂钓、海草房等特色项目和地方旅游产品的开发，把旅游观光、海鲜餐饮、民俗文化演艺和特色产品购物等有机结合起来，相互促进，力争旅游总收入增长15%以上。二是积极培育新的发展亮点。坚持市场化运作，高水平办好第四届国际人居节、中国首届轮胎博览会、第二届国际水产品博览会等展会，推动会展业与商贸、旅游等产业的融合发展。大力发展商贸物流业，加快威海中韩商品物流贸易批发中心、中国工艺家纺城、石岛北方渔市等市场建设。继续优化金融生态环境，强化资本监管和风险控制，推动金融业发展和创新。加快发展信息咨询、社区服务和中介组织，拓宽现代服务业的发展领域。三是进一步优化发展环境。坚持“非禁即入”，建立公开、平等、规范的行业准入制度，尽快引进和培植一批规模大、知名度高的名牌服务企业，争取在提高服务业比重上有大的突破。

围绕以海产品为主的农副产品出口加工业基地建设，积极发展富有特色的现代农业。继续加大各项惠农政策扶持力度，加强农业标准化体系建设，在稳定粮食产量的基础上，引导农民按照市场需求大力发展特色种植业和养殖业，年内调整经济作物20万亩。加快发展现代海洋产业，支持企业搞好无公害产地认证，建设现代渔业、生态渔业和品牌渔业。健全农产品质量安全监测体系，加大动植物疫病的防治力度。大力扶持各类农村合作经济组织和农民专业协会，搭建农民与政府、农民与市场、农民与技术之间的桥梁，提高抵御市场风险的能力。支持农业龙头企业做大做强，积极引进国内外先进的加工企业和技术，保持农产品出口稳定增长。争取农产品出口增长15%以上。

（三）保持消费和投资协调增长。正确处理消费和投资的关系，合理控制投资增长，努力扩大消费需求，推动经济增长逐步由“投资推动型”向“消费拉动型”转变。

一是积极扩大消费需求。引导企业在生产发展的同时，增加职工工资，提高城乡居民特别是中低收入者的收入水平，增强居民消费能力。认真实施市区商业网点规划，抓好城里中学原址、老汽车站和威韩商城等区域的规划建设，引进名企名店，整合商业资源，提升中心商业区的层次。积极开发市场，引导需求，鼓励文化、娱乐、健身等方面的消费，进一步繁荣晚间市场和休闲经济。推进“社区商业双进工程”和“万村千乡市场工程”，加快农村社区综合服务中心建设，引导连锁超市向社区、农村延伸，新建“农家店”500家。整顿和规范市场秩序，严厉打击假冒伪劣产品和市场欺诈行为，营造诚信、公平的消费环境。

二是努力优化投资结构。坚持以国家调控政策为导向，综合运用土地、环保、财政、信贷和产业政策等手段，强化对投资项目的源头管理，引导投资向自主创新、节能降耗、环境保护领域倾斜，向高新技术产业、重大装备、先进制造业倾斜，向农业农村、现代服务业、社会事业倾斜。集中力量抓好威乳高速及疏港路、环海生态旅游示范路、威海船厂整体搬迁等70个市级重点项目建设。深入实施“以港兴市”战略，抓好威海港三四期、客运中心搬迁等工程。完成机场停机坪改造等工程，进一步提高机场吞吐能力。积极做好青烟威荣城际铁路、中韩铁路轮渡、乳山红石顶核电站、昆嵛山抽水蓄能电站等项目的前期工作。充分发挥国资公司等融资平台的作用，加强与各金融机构的合作，进一步活跃民间资本，多渠道筹集资金。全年完成规模以上固定资产投资800亿元。

（四）进一步提高全方位开放水平。适应WTO过渡期结束后的新形势，深入实施互利共赢的开放战略，加快构建全方位、宽领域、高层次的开放格局。

一是努力提高利用内外资的质量。继续把引进大项目、好项目作为招商引资的重中之重，在稳定韩日投资的同时，加大对欧美、台港澳和东南亚的开拓力度，扩大与长三角、珠三角、京津塘地区及东北、西北城市群的合作，精心组织好赴美国、韩国、日本和上海、西安、哈尔滨等国内外经贸推介活动。突出产业和基础设施招商，积极推进镆铘岛中石油、三星造船、温泉开发及五星级酒店等项目的引进工作。坚持引资与优化结构相结合，鼓励企业与国内外大企业开展合资合作，努力引进一批先进技术、先进设备和研发中心。

二是加快转变外贸增长方式。积极调整出口产品结构，进一步扩大机电产品、高新技术产品和拥有自主知识产权产品的出口份额，支持纺织、农产品出口企业加大市场开拓力度，增强产品竞争力。大力推动加工贸易升级转型，引导企业由加工装配向自主研发制造延伸，提高加工深度和本地原材料采购比重。积极运用国际通行规则，建立和完善产业损害预警监测机制，提高企业应对和化解贸易摩擦的意识和能力。抓住国家大力支持进口的有利时机，增加我市急需的先进技术、关键设备、重要资源的进口。全年进出口总值增长16%以上。

三是着力增强园区集聚功能。高标准搞好现有省级以上开发区建设，提高项目的进区门槛，提升投资率和投资回报率，引导新上项目向各类园区集聚，加快形成群体优势和集聚效应。充分发挥两个国家级开发区和出口加工区的龙头带动作用，争取在引进大项目、高新技术项目上实现新突破。抓好威海工业园建设，强化基础设施配套和选商择资，努力打造现代制造业新区。

四是积极实施“走出去”战略。支持有条件、有信用的企业到境外投资，开发紧缺资源，建立生产和销售基地，扩大境外工程承包和劳务合作，重点抓好成山老挝橡胶园、三角印尼橡胶园、好当家菲律宾开发养殖、国际合作公司刚果铁路工程等项目。按照上级要求，积极做好重点帮扶和对口支援工作。

（五）积极推进关键领域的改革。围绕解决发展中的深层次矛盾和问题，继续搞好体制机制创新。

一是深化国有资产管理体制改革。健全国有企业负责人激励约束机制、重大事项监督管理机制和国有资本经营计划管理机制，盘活调优做强国有资产。加强国有资产收益管理，理顺机关事业单位资产经营收益的管理机制，实现国有资产保值增值。

二是健全完善公共财政体制。完善税收征管体制，推进社会综合治税，强化非税收入管理，提高税收质量和效益，确保财税“三个比重”有新的提高。继续深化部门预算、财政投资评审、国库集中支付和政府采购改革，加强对政府投资项目的审计监督，强化预算约束，规范投资行为，提高资金使用效益。

三是继续深化企事业单位改革。以建立现代企业制度为重点，突出抓好困难企业改制和解困工作。抓住资本市场发生转折性变化的有利时机，大力培植上市资源，支持华东数控、蓝星玻璃、工友等企业上市。继续深化农村信用社改革，做好央行票据兑付工作。加大事业单位改革步伐，全面推行人员聘用制。认真完成国铁重组我市地方铁路的各项工作，提高我市铁路运输能力。

四是大力发展民营经济。认真落实鼓励支持民营经济发展的一系列政策措施，加快实施中小企业成长计划，进一步完善融资担保、信用评价、人才培训、技术信息、法律服务和行业协会等服务体系，努力营造鼓励创业、宽容失败的发展环境。民营经济增加值增长18%以上。

四、加快构建社会主义和谐社会

坚持以人为本、以民为先，着力解决事关群众切身利益的问题，推动经济社会、城乡区域、人与自然的和谐发展。

（一）扎实推进社会主义新农村建设，促进城乡协调发展。坚持把解决“三农”问题作为加快构建社会主义和谐社会的重点，在发展农村生产力的基础上，逐步解决城乡发展不均衡的问题。

一是切实加快农村基础设施建设和公益事业发展。进一步提升完善村村通油路工程和村村通自来水工程，改善农村交通条件，保障农村饮水安全。大力发展节水灌溉，搞好坤龙邢、院里、米山等大中型水库除险加固，加强小流域综合治理。完善县级电网主网架，推进电气化镇村建设。加快镇卫生院、中心卫生室的上档升级建设，选择436个有条件的村，增设体育健身设施。继续实施“百村示范、千村整治”工程，搞好文明生态村创建活动。

二是积极推进农村综合改革。深化县乡财政管理体制改革，加快建立农村投入稳定增长机制，进一步完善激励性转移支付政策，切实增强县乡财政保障能力。坚持农村“一事一议”制度，控制农资价格，防止农民负担反弹。认真落实中央提出的“五年内乡镇机构编制只减不增”的要求，加大镇村撤并力度，推进镇级机构改革。

三是严格加强耕地保护。坚持农村基本经营制度，规范土地流转秩序。搞好土地利用总体规划修编工作，落实耕地保护目标责任制，确保基本农田总量不减少、用途不改变、质量不降低。大力实施土地开发整理复垦，搞好城镇建设用地增加和农村建设用地减少挂钩试点工作。加强和改进征地管理，严格执行征地补偿实名制，确保补偿费及时足额到位，切实维护农民利益。

四是大力培养新型农民。深入开展“三下乡”活动和“科普惠农行动”，大力实施“新农村建设农业科技行动计划”，提高农民务农致富和务工就业的能力。抓好新型农民科技培训和农民劳动力转移培训，全年培训转移农村劳动力2万人。加强农村文化设施建设，巩固农村文化阵地，提高农民的思想文化素质和农村的文明程度。

（二）推进生态市和精品城市建设，促进人与自然和谐发展。全面实施《威海生态市建设总体规划》，扎实推进生态市、生态县、环境优美乡镇建设，争取在全省率先基本建成生态市。严格执行环境影响评价和“三同时”制度，着力推进规划环评，从源头上控制污染。切实加强对自然保护区、水源涵养区、重点河流、近岸海域等区域的保护，加快编制集中式饮用水源地保护规划，解决好水源地流域内的各类污染和非法采砂问题。加快推进“数字环保工程”，建立健全环境监测预警体系。加强机动车尾气、噪音污染的专项治理，完成威海热电厂等7家企业的二氧化硫污染治理任务，抓好市第二污水处理厂和各工业园区的小型污水处理设施建设，完成艾山垃圾厂固体废弃物综合处理利用工程，城市污水集中处理率提高1个百分点。大力搞好植树造林，加强森林防火和林业有害生物防治，抓好威乳高速公路、威石线和乳山7.5万亩荒山绿化二期等重点工程，确保年内植树2000万棵以上。

坚持高起点规划、高标准建设、高效能管理，加快推进精品城市建设。继续搞好新一轮城市规划编制，抓好专项规划与城市总体规划的衔接。推进威海新外滩、金线顶公园、环翠楼公园改造等工程，建设一批街头和小区的小游园、小广场。完成烟威天然气长输管道建设和市区燃气管道配套，新增天然气用户8000户。加快49个城中村改造步伐，实施塔山中路等19条道路的新建或改造工程，在侨乡广场等交通流量大的区域建设过街天桥或地下通道。市区新增和更新公交车300辆。健全完善城市管理长效机制，加大行政执法力度，搞好背街小巷、繁华街区、城郊结合部的综合整治。继续开展“入社区、解民忧”活动，搞好文化路等4条路段的排水工程，妥善解决集中供热、物业管理等方面的问题。

（三）千方百计扩大就业，完善社会保障体系。一是做好就业再就业工作。坚持城乡统筹，全面落实各项就业扶持政策，完善职业培训和就业服务体系，完成中心市区人力资源市场和技能实训中心建设，年内新增城镇就业5万人。深入开展创建“劳动关系和谐企业”活动，规范企业用工和职业介绍行为，进一步提高劳动合同签约率；加强劳动执法监察，严格执行最低工资保障、工资指导线和农民工工资保证金制度，严厉查处恶意拖欠工资行为，切实保障劳动者合法权益。

二是健全完善社会保障体系。继续推进城镇医疗保险全覆盖试点，做好外资企业、私营企业职工和农民工参保工作，进一步扩大社会保险覆盖面，养老、医疗、工伤保险人数分别增加2万人、3万人和3.5万人。加强各类社会保障基金监管，确保基金安全和保值增值。完善农村社会养老保险和失地农民生活保障制度。深入实施安康居住工程，加大中低价位、中小户型的普通商品住房开发力度，扩大住房公积金覆盖面，完善廉租住房制度，健全住房保障体系。年内新开发经济适用住房28.7万平方米。

三是加强社会救助体系建设。全面落实对困难群众的医疗、教育、住房等专项救助政策，进一步扩大城乡最低生活保障覆盖面，确保补助资金及时到位。大力发展社会福利事业，加强红十字会和慈善总会等组织建设。大力发展老龄事业，抓好公共养老设施建设，做好残疾人的康复、就业、奔小康工作。

（四）加快发展教育卫生事业，努力促进教育医疗公平。强化义务教育的政府保障责任，使公共财政向教育倾斜、教育资源向农村倾斜。调整优化农村中小学布局，不断改善办学条件。继续落实城乡贫困家庭学生免课本费、补助寄宿费和班车费等政策，妥善解决外来务工人员子女就学问题。威海城区新建和扩建校舍2万平方米，进一步缓解城区中小学班额过大问题。大力发展职业技术教育，把威海职业技术学院建成国家示范性高等职业院校。重视发展成人教育、继续教育、特殊教育、学前教育，鼓励发展各类民办教育。继续完善校地共建机制，大力支持驻威各高校的发展。

坚持医疗卫生的公益性质，努力改善城乡医疗服务条件。高度重视食品、药品安全，切实加强药品价格和医疗收费监管，整顿和规范医疗服务秩序。大力发展社区卫生服务，提高新型农村合作医疗的筹资标准和保障能力，努力缓解看病难、看病贵的问题。进一步加强公共卫生体系建设，提高疾病预防控制和突发公共卫生事件救治能力。完善计划生育利益导向机制，继续稳定低生育水平，推进人口现代化。

（五）繁荣文化体育事业，促进精神文明建设。大力发展文化事业和文化产业，抓好新闻出版、广播影视、网络服务、动漫产业的发展，活跃文化艺术创作，打造城市文化品牌。加快市民文化中心等工程建设，完成威海剧院改造，完善和强化市艺术馆、图书馆的功能。广泛开展群众性文化活动，繁荣广场文化、社区文化、企业文化和校园文化，挖掘、保护和利用好优秀历史文化遗产。充分发挥现有体育场馆作用，继续补充完善全民健身设施，积极开展全民健身活动，吸引更多国内外重大赛事来威举办。

以文明威海建设为核心，深入推进社会主义精神文明建设。加强社会主义核心价值体系与和谐文化建设，大力弘扬威海精神，倡导爱国、敬业、诚信、友善等道德规范，广泛开展以“讲文明、促和谐”为主题的群众性精神文明创建活动。搞好新一轮地方史志编修和档案“社会记忆工程”。做好“双拥共建”工作，加强民兵预备役和人民防空建设。进一步做好妇女儿童、民族、宗教、外事、侨务、对台、气象、防震减灾等其他各项工作。

（六）加强民主与法制建设，构建平安和谐威海。进一步完善居民和村民自治，切实组织好村委会换届选举，强化街道和居委会的职能，加快“村改居”步伐，推进城市社区建设。认真实施依法治市五年规划，大力推进“五五”普法。健全三级法律援助网络，加强各类调解组织建设，加快仲裁事业发展，完善防范化解社会矛盾的长效机制，妥善处理征地、拆迁、环保、企业重组改制和破产等工作中出现的各类矛盾，切实维护群众的合法权益。继续完善各类应急管理体制，加强专业应急队伍和应急信息平台建设，提高危机管理和抗风险能力。开展“安全生产法规落实年”活动，严格落实安全生产责任制，杜绝特大恶性责任事故。强化社会治安综合治理，深入开展农村平安建设，完善人防、物防、技防、心防体系，不断深化严打整治斗争，确保社会稳定。

各位代表！构建和谐威海，是全市人民的最大福祉，也是各级政府执政为民的根本体现。我们一定要始终把人民群众的利益放在首位，以和谐的理念认识问题，以和谐的态度对待问题，以和谐的方式处理问题，把为民办实事贯穿于各个领域、各项工作、各个环节，坚持不懈地为民解忧、为民谋利、为民造福，使市政府的各项工作真正体现群众意愿，符合群众利益，满足群众需求。

五、在管理创新中努力推进政府自身建设

面对新的形势，我们一定要不断提高依法行政、科学行政、民主行政的能力和水平，以管理创新、廉洁高效为目标，加快建设法制政府、服务政府、责任政府和效能政府。

坚持以人为本、执政为民，增强政府的凝聚力。深入实施《公务员法》，全面加强公务员队伍建设。继续办好行风热线、直播威海，完善政府公报和政府网站，深化民主评议行风工作，充分发挥市长公开电话、市长信箱和社情民意中心的作用，密切同人民群众的联系。大力推进电子政务建设，积极扩大政务公开，将行政许可、行政收费、工程招标、土地使用等重要事项，严格置于群众的监督之下，切实保障人民群众的知情权、参与权和监督权。

坚持转变职能、依法行政，增强政府的公信力。推进政企、政资、政事、政府与中介组织分开，加强各级行政审批服务中心和便民服务中心建设，进一步减少和规范行政审批事项，把更多的精力用于公共服务和社会管理。严格按照法定权限和程序行使权力，自觉接受人大、政协和社会各界的监督。认真落实行政执法责任制和执法过错追究制，坚决防止行政“不作为”和“乱作为”。完善公众参与、专家论证和政府决策相结合的决策机制，提高科学民主决策水平。加强诚信政府建设，言必行、行必果，有诺必践，取信于民。

坚持改进作风、狠抓落实，增强政府的执行力。加强重大决策事项督查，完善政府部门沟通、协调、配合机制，确保政令畅通。转变工作作风，倡导求真务实，坚决反对形式主义和虚报浮夸，不提脱离实际的目标和口号，不搞劳民伤财的“政绩工程”和“形象工程”。围绕落实科学发展观、构建和谐社会，分解目标，强化责任，抓好政府各项工作的落实。

坚持从严治政、廉洁从政，增强政府的感召力。按照中央的要求，把反腐倡廉工作融入经济建设、政治建设、文化建设和社会建设之中，积极构建防范、惩治、保障和自律机制，从源头铲除滋生腐败的土壤和条件。严格落实“一岗双责”，自觉遵守廉政建设各项规定，充分发挥审计、监察部门的职能，进一步强化对经济领域重点事项的预防性监督，严肃查处工程建设、土地出让、产权交易、医药购销等重点领域的商业贿赂案件，坚持用制度管人、管权、管事，形成防治腐败的长效机制。

各位代表！我们正处在一个重要的发展时期，既面临着又好又快发展的繁重任务，又肩负着富民强市的崇高使命。让我们高举邓小平理论和“三个代表”重要思想伟大旗帜，紧密团结在以胡锦涛同志为总书记的党中央周围，在市委的正确领导下，坚持以科学发展观统领经济社会发展全局，同心同德，扎实奋进，开拓创新，为全面完成今年的各项目标任务、开创威海更加美好的未来而努力奋斗！
